# Vitamin K2 supplementation and the progression of abdominal aortic calcification in dialysis patients

**DOI:** 10.20407/fmj.2020-020

**Published:** 2020-12-16

**Authors:** Shoya Oyama, Naoki Okamoto, Shigehisa Koide, Hiroki Hayashi, Shigeru Nakai, Kazuo Takahashi, Daijo Inaguma, Midori Hasegawa, Hiroshi Toyama, Satoshi Sugiyama, Yukio Yuzawa, Naotake Tsuboi

**Affiliations:** 1 Department of Nephrology, Fujita Health University, School of Medicine, Toyoake, Aichi, Japan; 2 Faculty of Clinical Engineering, Fujita Health University, School of Medical Sciences, Toyoake, Aichi, Japan; 3 Department of Biomedical Molecular Sciences, Fujita Health University, School of Medicine, Toyoake, Aichi, Japan; 4 Department of Radiology, Fujita Health University, School of Medicine, Toyoake, Aichi, Japan; 5 Kanayama Clinic, Nagoya, Aichi, Japan

**Keywords:** Vitamin K, Aortic abdominal calcification, Dialysis, Randomized control trial, Agatston score

## Abstract

**Objectives:**

Vascular calcification is common in patients with advanced chronic kidney disease (CKD) and contributes to cardiovascular disease. Accumulating evidence indicates that CKD patients often acquire subclinical vitamin K deficiency, which is associated with vascular calcification.

**Methods:**

This prospective, randomized, parallel group, multicenter trial (UMINID000011490) will include 200 dialysis patients in an open-label, two-arm design. After baseline computed tomography of the abdominal aorta, patients will be randomized to two groups that will either (1) continue receiving standard care or (2) receive additional oral supplementation with menatetrenone (45 mg/day). The treatment duration will be 24 months, and the computed tomography scan will be repeated after 12 and 24 months. The primary endpoint is the progression of abdominal aortic calcification, which is calculated as absolute changes based on the Agatston score. The secondary endpoints are the decrease in bone mineral density (measured by dual-energy X-ray absorptiometry), the biomarkers associated with vitamin K, vitamin K intake (evaluated by the food frequency questionnaire), and the biomarkers associated with vascular calcification.

**Conclusions:**

This study aims to confirm whether vitamin K has inhibitory effects on calcification that can be clinically determined.

**Trial registration:**

UMINID000011490.

## Introduction

Patients with chronic kidney disease (CKD) have an extremely high risk of developing vascular disease,^1^ and vascular calcification is an independent risk factor for cardiovascular mortality.^2^ Thus, vascular calcification is important for the prognosis of CKD. Clinically, vitamin D and K deficiencies are known to be risk factors for uremic vascular calcification.^3,4^ Vitamin K comprises a group of fat-soluble vitamins that act as co-factors for γ-glutamylcarboxylase, which activates several vitamin K-dependent proteins. Matrix Gla protein is a vitamin K-dependent protein that is involved in inhibiting vascular calcification. The inactive form of matrix Gla protein reflects vitamin K deficiency and has been reported to be associated with cardiovascular events and mortality.^5^ Clinical and epidemiological studies have also indicated that long-term treatment with a vitamin K antagonist (warfarin) increases the risk of vascular calcification in certain individuals, possibly because of a poor vitamin K status.^6^ In CKD, vitamin K deficiency is highly prevalent and associated with vascular calcification and cardiovascular mortality.^7^ Vitamin K2 supplemented as menatetrenone is covered by insurance owing to its effects on osteoporosis in Japan. The aim of this clinical trial is to determine whether vitamin K2 supplementation has an inhibitory effect on vascular calcification in addition to osteoporosis in patients undergoing dialysis.

## Overall study aims

The primary endpoint is to determine whether vitamin K2 decreases the progression of aortic calcification based on absolute changes as per the Agatston score.

## Trial design

This study is a multicenter, randomized, prospective, open-label, interventional trial. Two treatments will be compared, providing a two-arm parallel group design with a total of 200 patients.

## Inclusion criteria

Patients with the following criteria will be included in the trial:

(1)  Males and females ≥18 years of age
(2)  Patients receiving hemodialysis or peritoneal dialysis
(3)  Patients diagnosed with osteoporosis based on bone mineral density, blood sampling results, or patient history.

## Exclusion criteria

(1)  Life expectancy <12 months
(2)  Pregnancy
(3)  Intake of vitamin K antagonists
(4)  Inflammatory bowel disease
(5)  Hypersensitivity to vitamin K
(6)  History of thrombosis
(7)  Severe liver dysfunction.

## Treatment details

All patients will first undergo a multi-slice computed tomography (CT) scan of the abdomen. We will use the Agatston score to quantify calcification. The Agatston score is a semi-automated tool used to calculate a score based on the extent of arterial calcification detected by an unenhanced CT scan. The calculation is based on the weighted density score given to the highest attenuation value (HU) multiplied by the area of the calcification speck (130–199 HU: 1; 200–299 HU: 2; 300–399 HU: 3; and 400+ HU: 4). The calcification of the abdominal aorta will be measured from the celiac artery branch to the inferior mesenteric artery branch. The aorta will be regarded as a perfect circle, and only calcifications inside the circle will be selected. Calcifications of the bifurcating arteries that branch from the abdominal aorta (celiac, bilateral renal, superior mesenteric, testis, ovarian, and inferior mesenteric arteries) will be excluded. Dialysis patients will be divided into two groups: a group administered with vitamin K2 and a control group. The hemodialysis and peritoneal dialysis patients will be randomly allocated into two groups. Half of the hemodialysis patients and half of the peritoneal dialysis patients will be administered with vitamin K2 (the vitamin K2 administration group), whereas the other halves will receive the standard care for CKD-mineral bone disease without vitamin K2 administration (the control group). The following seven factors will be considered.

Hemodialysis patients: age of 70 years, sex, time on dialysis of 10 years, diabetes, serum phosphate of 6.1 mg/dL, corrected calcium of 9.3 mg/dL, and a young adult mean of 70% or a T score of –2.5 in dual-energy X-ray absorptiometry.

Peritoneal dialysis patients: age of 64 years, sex, time on dialysis of 4 years, diabetes, serum phosphate of 6.1 mg/dL, corrected calcium of 9.3 mg/dL, and a young adult mean of 70% or a T score of –2.5 in dual-energy X-ray absorptiometry.

The observation period will be 24 months. After performing abdominal CT and dual-energy X-ray absorptiometry of bone density, an interventional study will be performed. In the vitamin K2 administration group, menatetrenone (45 mg/day) will be orally administered in three divided doses. Vitamin K-related biomarkers will be measured before the study and 2, 12, and 24 months later. Other calcification-related biomarkers will be measured before the study and 24 months later. All participants will answer the Food Frequency Questionnaire before the study. The abdominal CT and dual-energy X-ray absorptiometry of bone density will be repeated after 1 year and 2 years.

## Ethics approval and consent to participate

This study is being conducted according to the Ethical guidelines for Clinical Research by the Japanese Ministry of Health, Labour, and Welfare and the Helsinki Declaration. This study was approved by the ethics committee of Fujita Health University School of Medicine (approval number: HM20-153). The subjects will receive oral and written explanation of the study and will provide consent to be included in the study. The trial registration number is UMIN000011490.

## Primary endpoints

The primary endpoint is the progression of abdominal aortic calcification, which is calculated as absolute changes based on the Agatston score in 24 months. The period from baseline abdominal CT to the start of vitamin K2 administration will be less than 1 month.

## Secondary endpoints

We shall consider the following:

(1)  Bone mineral density: measured by dual-energy X-ray absorptiometry
(2)  Biomarkers associated with vitamin K
(3)  Vitamin K intake: evaluated by the Food Frequency Questionnaire
(4)  Biomarkers associated with vascular calcification
(5)  Progression of abdominal aortic calcification in 12 months.

## Justification of sample size

Sample size calculations were based on the studies by Brandenburg et al.^8^ and Krueger et al.^9^ Assuming the difference in the changes in the Agatston score between the two groups was 400 and the standard deviation was 900, we would need to enroll 80 patients per arm to detect a significant difference with a two-sided significance level of 5% and a detection power of 80%. Correcting our total sample size of 160 by a dropout rate of 20% resulted in a total sample size of 200.

## Analysis of the primary endpoint

The primary efficacy end point is the change in abdominal aortic calcification evaluated by the Agatston score at the 24-month visit versus the baseline visit. While we will use the change in the abdominal aortic calcification Agatston score after 24 months as an objective variable, the difference between the vitamin K administration and control group will be confirmed by covariance analysis. The abdominal aortic calcification Agatston score at the time of the baseline measurement will be added into the model as a covariate. The significance level will be set at 5% on both sides. If a Gaussian distribution will not be confirmed in the residuals, the change in the abdominal aortic calcification Agatston score will be examined after logarithmic transformation.

## Current registration status

Registration of this study started on April 21, 2014, and 110 cases have been registered. The number of participating facilities has increased to a total of 14, and we are planning to register the remaining 90 cases within 2 years.

## Discussion

The Agatston score is a coronary artery calcium score that was proposed by Agatston et al.^10^ in 1990. It has also been applied for the evaluation of aortic calcification. Since the purpose of this study is to verify whether vitamin K2 as a drug has an inhibitory effect on calcification, the progression of abdominal aortic calcification evaluated by the Agatston score will be set as the primary endpoint.

Few interventional studies examining the inhibitory effect of vitamin K on calcification have been performed. Brandenburg et al. reported that the aortic valve calcification volume score progressed by 10% in patients administered with vitamin K1 compared with 22% in a placebo group, representing a significant vitamin K1-induced attenuation of aortic valve calcification.^8^ Furthermore, Shea et al. reported that phylloquinone supplementation slowed the progression of coronary arterial calcification in healthy older adults.^11^ There have been two ongoing vitamin K interventional studies in dialysis patients in which vitamin K1 was administered to the interventional group. To date, there has been no vitamin K2 (menaquinone) interventional trial in dialysis patients. We believe our present trial will contribute to finding a treatment option for vascular calcification in dialysis patients.

Vitamin K-related markers are not routinely measured in maintenance dialysis patients, and the treatment for vitamin K deficiency has not been standardized yet. Therefore, we believe that not administering vitamin K supplementation for 2 years is not unethical.

The severity of vascular calcification in dialysis patients is higher than that in healthy subjects. Jansz et al. reported that the degree of coronary calcification was not significantly different between hemodialysis and peritoneal dialysis patients.^12^ We have assessed that similar treatment effects of vitamin K can be evaluated in hemodialysis and peritoneal dialysis patients. As patients use their own peritoneum, deterioration of peritoneal function hinders the continuation of peritoneal dialysis. Therefore, the time on dialysis is shorter in peritoneal dialysis patients than in hemodialysis patients. Patients must change the peritoneal dialysis bag by themselves, and the mean age of the peritoneal dialysis patients is younger than that of hemodialysis patients. For these reasons, the allocation factors differ between hemodialysis and peritoneal dialysis.

Although this is an open-trial test, we postulate that physicians will not change the standard care according to the allocation result because vitamin K administration is independent of routinely performed CKD-mineral bone disease treatment.

This study has some limitations. First, the long-term effects on cardiovascular prognosis cannot be evaluated because the observational period is 2 years. Second, instructions for returning any unused medication and empty medication containers are not included in the protocol.
